# Methamphetamine Administration Targets Multiple Immune Subsets and Induces Phenotypic Alterations Suggestive of Immunosuppression

**DOI:** 10.1371/journal.pone.0049897

**Published:** 2012-12-05

**Authors:** Robert Harms, Brenda Morsey, Craig W. Boyer, Howard S. Fox, Nora Sarvetnick

**Affiliations:** 1 Department of Surgery-Transplant, University of Nebraska Medical Center, Omaha, Nebraska, United States of America; 2 Department of Pharmacology and Experimental Neuroscience, University of Nebraska Medical Center, Omaha, Nebraska, United States of America; 3 Regenerative Medicine Project, University of Nebraska Medical Center, Omaha, Nebraska, United States of America; Temple University School of Medicine, United States of America

## Abstract

Methamphetamine (Meth) is a widely abused stimulant and its users are at increased risk for multiple infectious diseases. To determine the impact of meth on the immune system, we utilized a murine model that simulates the process of meth consumption in a typical addict. Our phenotypic analysis of leukocytes from this dose escalation model revealed that meth affected key immune subsets. Meth administration led to a decrease in abundance of natural killer (NK) cells and the remaining NK cells possessed a phenotype suggesting reduced responsiveness. Dendritic cells (DCs) and Gr-1^high^ monocytes/macrophages were also decreased in abundance while Gr-1^low^ monocytes/macrophages appear to show signs of perturbation. CD4 and CD8 T cell subsets were affected by methamphetamine, both showing a reduction in antigen-experienced subsets. CD4 T cells also exhibited signs of activation, with increased expression of CD150 on CD226-expressing cells and an expansion of KLRG1^+^, FoxP3^−^ cells. These results exhibit that meth has the ability to disrupt immune homeostasis and impact key subsets of leukocytes which may leave users more vulnerable to pathogens.

## Introduction

Methamphetamine (Meth) is a highly addictive psychostimulant and neurotoxicant of increasing popularity among drug-abusing populations worldwide [1]–[9]. This rising popularity has significant economic consequences, as in the US alone, the financial burden of meth use was calculated at >23 billion dollars annually [10]. Meth is typically administered nasally, intravenously or orally, and meth users experience feelings of euphoria, hyperactivity, reduced appetite, sleeplessness, and arousal after administration [11]. After injection, meth, a lipid-soluble monamine, has been shown to disseminate and accumulate widely throughout tissues in both humans and rats [12], [13], and there is an extensive body of data that describes the toxic effects of meth on the CNS and resulting neurologic damage and cognitive impairment [14], [15]. Meth users are prone to increased rates of several types of infections, including human immunodeficiency virus (HIV), hepatitis A, B, and C, and methillicin-resistant *Staphylococcus aureus*, due to their involvement in risky sexual practices and from the inherent dangers associated with intravenous drug use [16]–[23]. In addition to these behaviors putting users at increased risk of transmission of infectious agents, meth has been shown to promote HIV infection of human macrophages and increase hepatitis C replication within hepatocytes [24], [25].

Meth itself is an immunomodulatory substance with a wide range of effects that have been observed in diverse *in vitro* systems as well as murine and nonhuman primate models. Several lines of evidence support the notion that meth suppresses the immune system. Meth has been associated with reduced leukocyte proliferation [26]–[28], reduced IL-2 production [28]–[30], reduced immunoglobulin production [30], [31], and reduced macrophage and dendritic cell (DC) function [32]–[34]. Meth also promotes susceptibility to viral and fungal pathogens among hosts [27], [34], [35]. While meth appears to suppress the response of B and T cells, macrophages, and DCs, studies have shown that natural killer (NK) cells exhibit increased levels of activation after meth exposure [29], [30], [35], [36], although at least one study reported reduced NK cell function [37]. Meth has also been observed to alter immune function in the brain, with increased microglia activation and abundance after meth exposure [38]. Finally, meth has been reported to promote apoptosis in the thymus and spleen [39], as well as among cultured T cells [28].

Taken together, these studies reveal that meth has the ability to profoundly interfere with immunological networks, affecting diverse leukocyte subsets and thereby leaving the user vulnerable to pathogens. Although several studies on meth have employed flow cytometry to evaluate the immune response, this has not been performed in a comprehensive manner to elucidate specific cellular alterations induced by meth. Indeed, multiparameter flow cytometry can be used to generate a detailed analysis of the expression level of multiple proteins at the single cell level. This allows the investigator to generate a complex, yet more complete, picture of immunological networks in states of health, disease, and toxicant exposure, and ultimately produce a wealth of observations to guide further investigations. As previous studies suggested profound and detrimental impacts of meth on T cell, NK cell, and macrophage/DC responses, we hypothesized that these deficiencies would result in specific phenotypic alterations of these cells, thereby suggesting altered functionality *in vivo*. To explore this hypothesis, we treated C57BL/6 mice with levels of methamphetamine that simulate usage patterns observed in typical human addicts (following methods in [34]) and then examined multiple leukocyte subsets to observe how meth changes the phenotypic appearance of immune cells. Our results reveal that meth treatment reduces the overall abundance of activated/antigen-experienced CD4 and CD8 T cells while promoting activation and expansion of discrete CD4 T cell subsets. We also observed that meth impacts splenic myeloid cells, reducing the number of DCs and Gr-1^high^ monocytes/macrophages, while promoting the perturbation of Gr-1^low^ monocytes/macrophages. Finally, we observed that meth contributes to a reduction in number of splenic NK cells and leaves the majority of remaining NK cells with a phenotype suggesting reduced responsiveness and functional efficacy.

## Methods

### Mice and Meth Treatment

Male C57BL/6 mice 12–14 weeks of age were purchased from Jackson Labs, housed in specific pathogen free conditions, and given unlimited access to food and water. We purchased (+)-methamphetamine hydrochloride from Sigma-Aldrich and employed a treatment regimen similar to that described in [34] that simulates meth usage patterns of typical human addicts. Meth was administered via intraperitoneal injections in a ramped dosing schedule on days 1–4 with 2, 4, 6, and 8 mg/kg given per day. On days 5–14, we plateaued the dosage to 10 mg/kg/day. Control mice were given saline solution (vehicle) over this same time period. On day 14, within 3 hours of the final injection, mice were anesthetized with isofluorane and euthanized via cervical dislocation. The Institutional Animal Care and Use Committee of the University of Nebraska Medical Center approved all procedures.

### Flow Cytometry

Spleens and mesenteric lymph nodes were harvested from freshly euthanized mice. These were then teased apart with fine forceps and ran through nylon mesh screens to produce single cell suspensions. Red blood cells (RBCs) were removed from splenocyte preparations by utilizing ammonium chloride RBC lysis buffer. Prior to labeling cells with antibodies, we stained cells with Live/Dead^©^ Fixable.

Blue Dead Cell Stain (Life Technologies) and then blocked Fc receptors using unlabeled, irrelevant rat IgG. We used mouse monoclonal antibodies from multiple sources in our flow cytometry panels. From BD Pharmingen, we utilized B220(RA3-6B2), CD3e(500A2), CD11c(HL3), CD27(LG.3A10), CD44(IM7), CD80(16-10A1), and NKp46(29A1.4). From eBiosciences, we used CD4(GK1.5), CD8(53-6.7), CD11b(M1/70), CD25(PC61.5), CD62L(MEL-14), CD94(18d3), CD86(GL-1), Gr-1(RB6-8C5), NKG2D(CX5), FoxP3(FJK-16s), MHC II(M5/114.15.2), Ly-49H(3D10) and Ly-49G2(eBio4D11). Antibodies against CD3(17A2), CD27(LG.3A10), CD45RB(C363-16A), KLRG1(2F1/KLRG1), CD150(TC15-12F12.2), and CD226(10E5) were purchased from BioLegend. For surface staining, cells were suspended in staining buffer consisting of phosphate buffered saline (PBS) containing 0.5% bovine serum albumin (BSA) and 0.05% sodium azide (NaN3). Surface-stained cells were fixed in 1.5% paraformaldehyde (PFA) for 1 hour, then washed and resuspended in staining buffer prior to analysis. For FoxP3 staining, we used FoxP3 Fixation/Permeabilization Concentrate and Diluent from eBiosciences according to the manufacturer’s recommendations. Cells were analyzed with an LSR II (BD Biosciences) within 24 hours of preparation.

### Analysis and Statistics

We used FlowJo (v. 8.8.6, TreeStar, Inc.) to analyze flow cytometry data. Values from FlowJo were transferred into GraphPad Prism (v. 4.0, GraphPad Software) for statistical analysis. To test for significant differences between meth-treated and control animals, we utilized the Mann-Whitney U Test.

## Results

### Meth Causes a Reduction in Absolute Number and Proportion of Splenic Natural Killer Cells, Gr-1(Ly-6C)^high^ Monocytes/macrophages, and DCs

**Figure 1:**
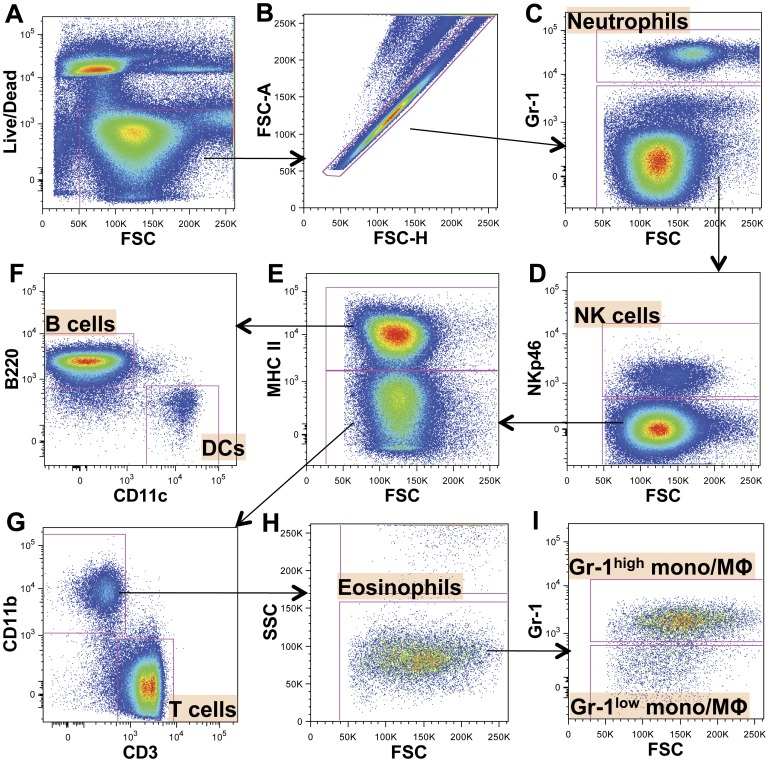
Gating strategy for defining major subsets of leukocytes among splenocytes. A. Dead cells were removed from the analysis using Live/Dead® fixable dead cell stain. **B.** Doublets were removed from living cells (Live/Dead^−^) using FSC-A and FSC-H. **C.** Gr-1^bright^ splenocytes were gated and defined as neutrophils. **D.** NKp46^+^ events were gated from Gr-1^low/−^ splenocytes and defined as natural killer (NK) cells. **E.** NKp46^−^, Gr-1^low/−^ splenocytes were divided into MHC II^+^ and MHC II^−^ populations. **F.** MHC II^+^ events were split by B220 and CD11c expression. MHC II^+^, B220^+^, CD11c^−^ events were defined as B cells. MHC II^+^ B220^−^, CD11c^+^ events were defined as dendritic cells (DCs). **G.** We split MHC II^−^ events by CD3 and CD11b expression. CD11b^-^, CD3^+^, MHC II^-^ events were labeled as T cells. **H.** CD11b^+^, CD3^−^, MHC II^-^ events were split according to SSC profile. Events with higher SSC values (suggesting greater internal granularity) were labeled as eosinophils. **I.** Events with lower SSC values were divided by Gr-1 expression, giving two populations: Gr-1(Ly-6C)^high^ monocytes/macrophages and Gr-1(Ly-6C)^low^ monocytes/macrophages (mono/MΦ).

To determine the impact of meth exposure on the proportion and number of splenic leukocyte subsets, we prepared splenocyte preparations for flow cytometric analysis with antibodies against CD3, B220, Gr-1, CD11b, CD11c, MHC II, and NKp46, allowing us to define and quantify T cells, B cells, natural killer (NK) cells, neutrophils, eosinophils, dendritic cells (DCs), and Gr-1^high^ and Gr-1^low^ monocytes/macrophages (see gating strategy Fig. 1 and Table 1). The Gr-1 antibody (clone RB6-8C5) binds to epitopes on Ly-6G and Ly-6C [40], and can be used to label neutrophils (SSC^med^, Gr-1^bright^ (co-expressing both Ly6G and Ly6C at high levels)) and divide splenic monocytes/macrophage into Gr-1^high^/^low^ subsets, corresponding to high or low levels of Ly-6C expression [41], [42]. NKp46 is an activating receptor specifically expressed by the NK cell lineage and can be used to effectively label these cells [43].

**Table 1:** Phenotype of splenic leukocyte subsets analyzed after meth treatment.

Leukocyte subset	Surface expression of relevant phenotypic markers
**T cells**	CD3^+^, MHC II^−^, Gr-1^−^, NKp46^−^, CD11b^−^
**B cells**	B220^+^, MHC II^+^, CD3^−^, Gr-1^−^, NKp46^−^, CD11c^−^
**Natural killer (NK) cells**	NKp46^+^, Gr-1^−^, MHCII^−^, CD3^−^
**Dendritic cells (DCs)**	CD11c^+^, MHC II^+^, CD11b^+/−^, CD3^−^, B220^−^, NKp46^−^
**Neutrophils**	CD11b^+^, Gr-1^bright^, SSC^med^, CD3^−^, B220^−^, CD11c^−^, MHCII^−^, NKp46^−^
**Eosinophils**	CD11b^+^, Gr-1^low^, SSC^high^, CD3^−^, B220^−^, CD11c^−^, MHCII^−^, NKp46^−^
**Gr-1(Ly-6C)^high^ mono/MΦ**	CD11b^+^, Gr-1^+^, MHCII^−^, CD3^−^, B220^−^, CD11c^−^, NKp46^−^
**Gr-1(Ly-6C)^low^ mono/MΦ**	CD11b^+^, Gr-1^−^, MHCII^−^, CD3^−^, B220^−^, CD11c^low/−^, NKp46^−^

**Figure 2:**
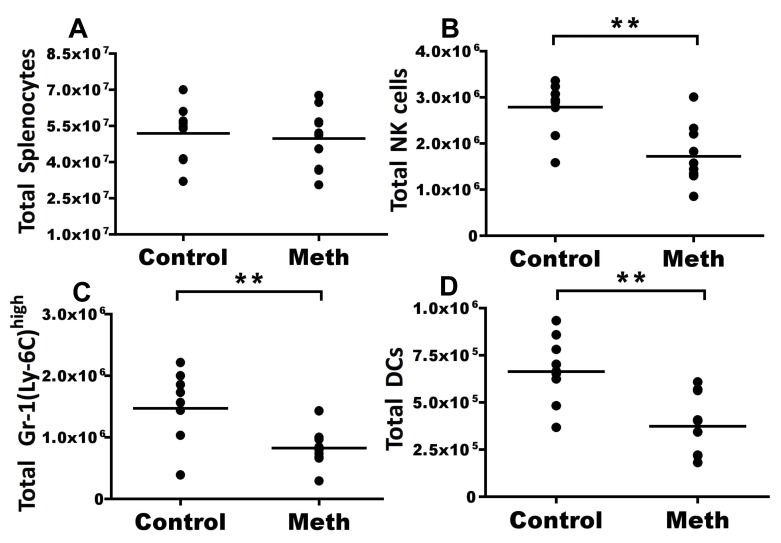
Meth causes a reduction of splenic leukocyte subsets. C57BL/6 mice were treated with vehicle (control) or ramped dosage of methamphetamine (meth – see methods) for 14 days. At day 14, mice were sacrificed and splenic leukocyte subsets were analyzed by flow cytometry. **A.** Meth treatment did not alter total splenocyte number. **B.** Meth causes a significant reduction in total natural killer (NK) cells (NKp46^+^, CD3^−^, B220^−^, Gr-1^−^, MHC II^−^). **C.** Gr-1^high^ monocytes (CD11b^+^, Gr-1^high^, MHC II^−^, CD3^−^, B220^−^) were reduced after meth treatment. **D.** Meth treatment reduces dendritic cells (DCs) defined as CD11c^+^, MHC II^+^, B220^−^, Gr-1^−^ events. Dead cells and doublets were removed prior to analysis. **P<0.01 calculated by Mann-Whitney U Test. Bars represent mean. Data are from 2 experiments of 5 animals per treatment group per experiment.

Our results indicated that although absolute counts of total splenocytes were not different between meth-treated and control groups (Fig. 2A), meth treatment caused significant decreases in proportion (data not shown) and number of NK cells (p = 0.003), Gr-1(Ly-6C)^high^ monocytes/macrophages (p = 0.0041), and DCs (p = 0.0015) in meth-treated mice (Fig. 2B–D). T cells, B cells, neutrophils, eosinophils, and Gr-1(Ly-6C)^low^ monocytes/macrophages exhibited no significant difference in proportion or absolute number after meth treatment (data not shown). These results show that meth has the ability to negatively impact the abundance of innate immune subsets.

### Meth Treatment Causes the Perturbation of Gr-1(Ly-6C)^low^ Monocytes/macrophages

**Figure 3:**
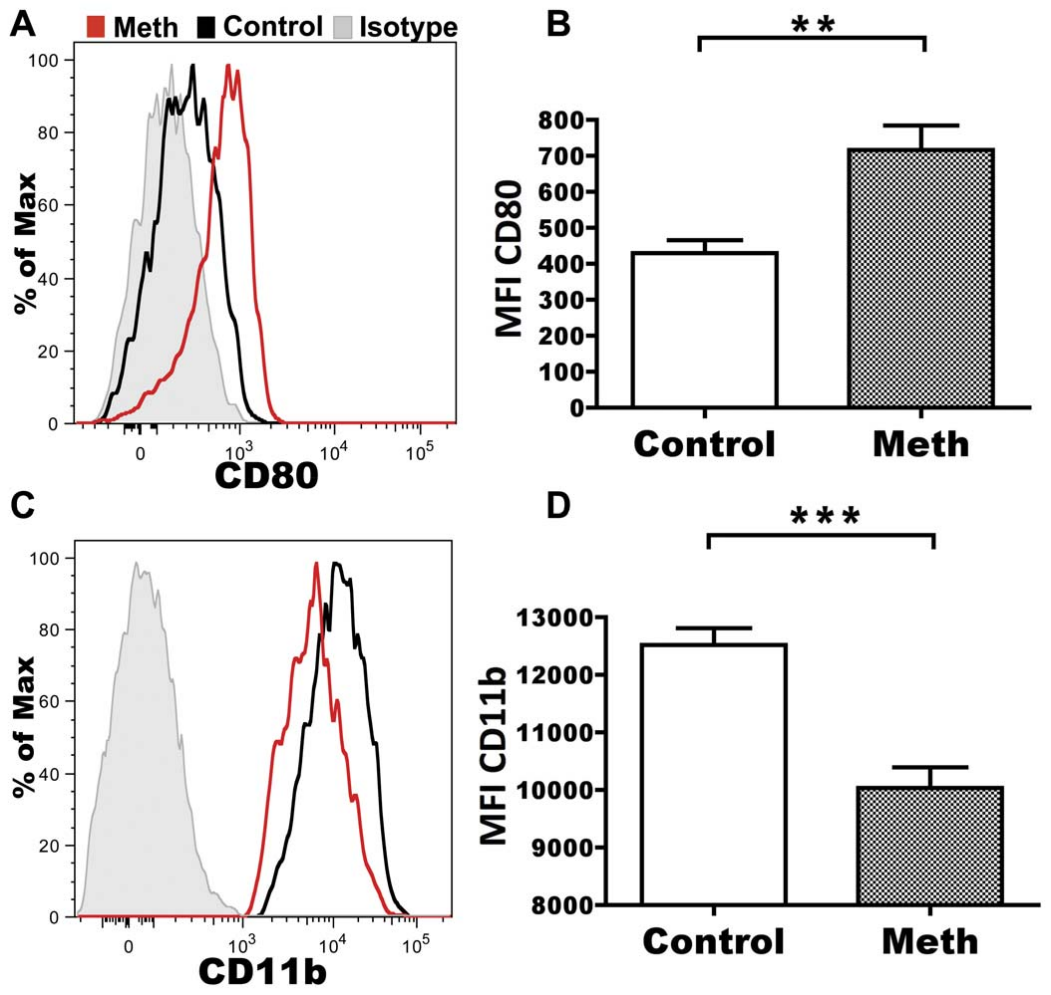
Meth perturbs Gr-1^low^ monocytes/macrophages. Gr-1^low^ monocytes/macrophages were analyzed for signs of activation after meth treatment using flow cytometry. **A.** Representative histogram showing CD80 (B7-1) expression by Gr-1^ow^ monocytes/macrophages. Red represents a meth-treated animal, black represents a vehicle-treated animal, and grey is the isotype control. **B.** Meth causes increased expression of CD80 by Gr-1^low^, quantified by mean fluorescence intensity (MFI). **C.** Representative histogram showing CD11b (Mac-1) expression by Gr-1^low^ monocytes/macrophages with colors as in **A** above. **D.** Meth causes decreased expression of CD11b by Gr-1^low^ monocytes, quantified by MFI. Dead cells and doublets were removed prior to analysis. **p<0.01, ***p<0.001 calculated by Mann-Whitney U Test. Error bars represent SEM. Data are from 2 experiments of 5 animals per treatment group per experiment.

Meth treatment has been shown to upset antigen presentation pathways and promote apoptosis, thus suggesting an alteration of antigen presenting cell (APC) activation with meth treatment. To investigate the role of meth treatment on APC and APC-precursor activation levels, we labeled splenic DCs and monocytes/macrophages with antibodies against the costimulatory markers CD80 and CD86 [44], and then examined surface protein expression by flow cytometry. Interestingly, Gr-1^low^ monocytes/macrophages showed statistically significant upregulation of CD80 (p = 0.0057; measured by mean fluorescence intensity (MFI)), but not CD86 (data not shown), after meth exposure (Fig. 3A & B). This increase in CD80 expression was coupled with a decrease in CD11b expression by Gr-1^low^ monocytes/macrophages (p = 0.0004; Fig. 3C & D). There was no change in CD80 or CD86 expression levels by Gr-1^high^ monocytes/macrophages and DCs (data not shown). An upregulation in CD80 in parallel with a downregulation of CD11b suggests that that Gr-1^low^ monocytes/macrophages are perturbed after meth treatment, while Gr-1^high^ monocytes/macrophages appear to be less sensitive to meth-induced activation.

### A Greater Proportion of NK Cells Possess a Phenotype Suggesting Terminal Differentiation After Meth Exposure

**Figure 4:**
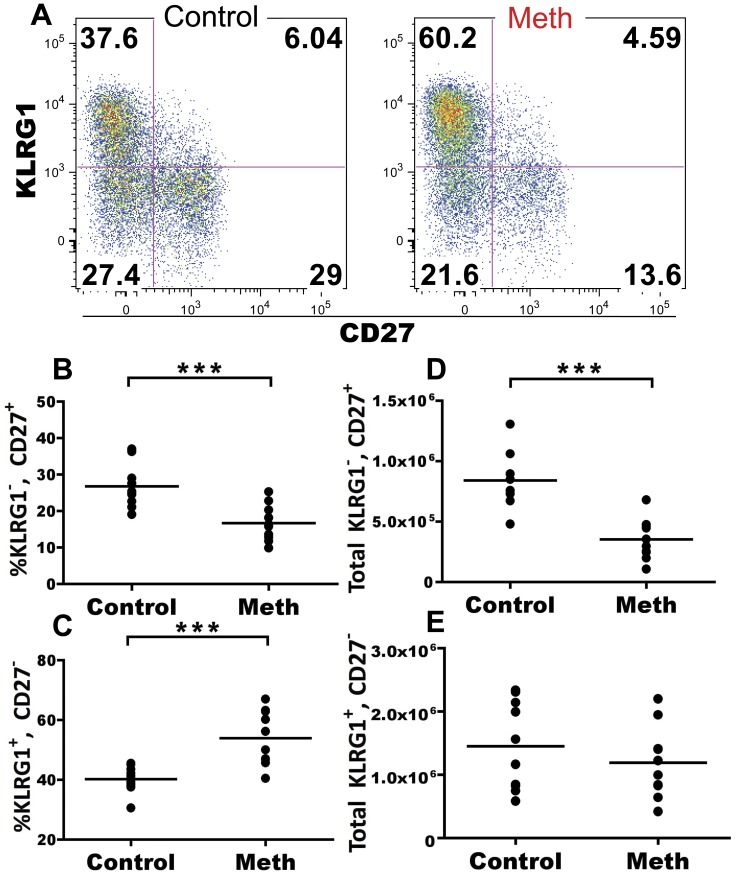
Meth alters NK cell subsets. We labeled splenic NK cells (NK1.1^+^, CD4^−^, CD8^−^) with antibodies against killer cell lectin-like receptor G1 (KLRG1) and CD27 to determine if meth treatment alters functionally distinct NK cell subsets. **A.** Representative gating showing KLRG1 and CD27 on gated NK cells. **B and C.** Meth treatment results in a decreased proportion of KLRG1^−^, CD27^+^ NK cells with an increase in proportion of KLRG1^+^, CD27^−^ NKs. **D and E.** Although proportionally higher, KLRG1^+^, CD27^−^ NK cells are not increased in absolute number. However, KLRG1^−^, CD27^+^ NK cells were significantly reduced in number. Dead cells and doublets were removed prior to analysis. ***p<0.001 calculated by Mann-Whitney U Test. Bars represent mean. Data are from 2 experiments of 5 animals per treatment group per experiment.

Meth treatment has been shown to both promote and inhibit NK cell activation. To address if NK cells exhibit an altered phenotype suggesting changes in maturation or development, we labeled NK cells (NK1.1^+^, CD4^−^, CD8^−^) with KLRG1, CD27, Ly-49H, Ly-49G2 and CD94. Among murine NK cells, CD27 expression is associated with naïve status of NK cells and KLRG1 expression is associated with terminal differentiation [45], [46]. We noticed a significant decrease in proportion of KLRG1^−^, CD27^+^ NK cells (p = 0.0011) concurrent with a significant increase in KLRG1^+^, CD27^−^ NK cells (p = 0.0002; Fig. 4A–C). While a greater proportion of NK cells possessed a terminally differentiated phenotype, this did not correspond to an overall increase in number. We observed that total KLRG1^+^, CD27^−^ NK cells were relatively similar between meth-treated and control animals (Fig. 4E). However, there was a significant decrease in total KLRG1^−^, CD27^+^ NK cells after meth treatment compared to control (p<0.0001; Fig. 4D). These results suggest that meth treatment may promote the terminal differentiation of NK cells and also reduce the abundance of non-terminally differentiated NK cells. No difference in Ly-49H, Ly-49G2, or CD94 expression was noted after treatment (data not shown).

### CD4 T Cells

#### Meth treatment results in decreased proportions of activated CD4 T cells in spleen and lymph node

**Figure 5:**
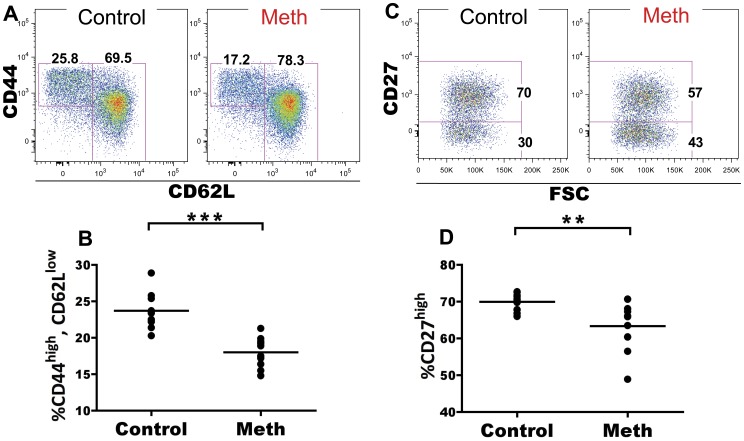
Meth reduces proportions of CD62L^low^, CD44^high^ splenic CD4 T cells and these cells exhibit lower CD27 expression. Splenic CD4 T cells were examined after meth treatment to determine if meth alters surface phenotypes suggesting activation/antigen experience and effector status. **A.** Representative gating showing CD62L and CD44 on CD4 T cells. **B.** Meth causes a reduction in proportion of CD44^high^, CD62L^low^ CD4 T cells. **C.** CD27 expression by CD44^high^, CD62L^low^ CD4 T cells. **D.** CD44^high^, CD62L^low^ CD4 T cells from meth treated animals exhibit a lower proportion of cells expressing CD27 at a high level. **p<0.01, ***p<0.001 calculated by Mann-Whitney U Test. Bars represent mean. Data are from 2 experiments of 5 animals per treatment group per experiment.

**Figure 6:**
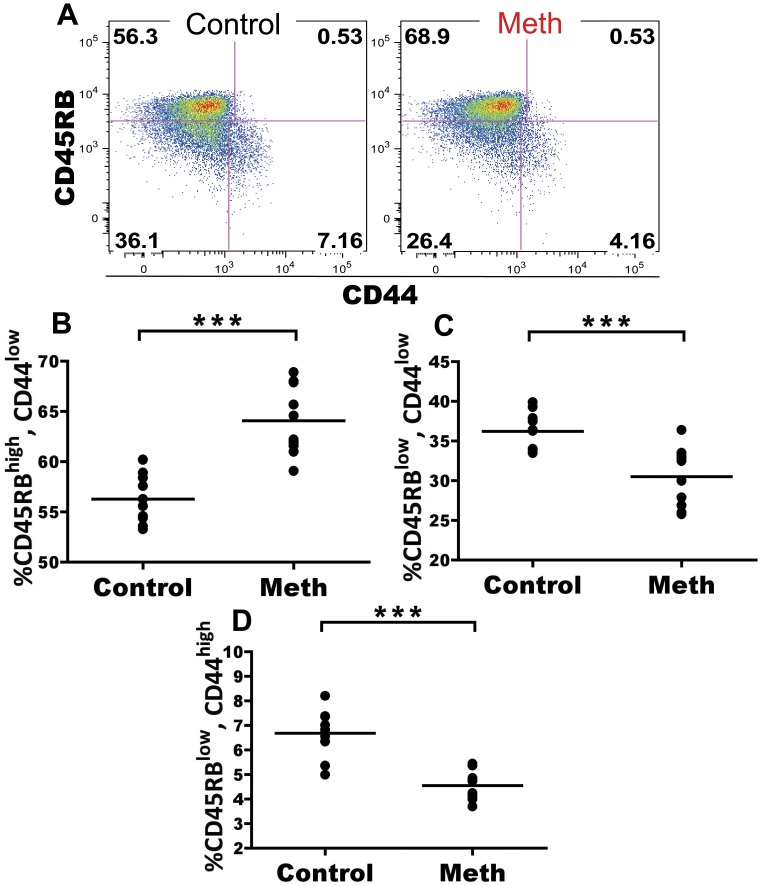
Meth causes reduced proportions of alternatively spliced CD4 T cells among the splenic CD62L^high^ compartment. Meth and control mice were analyzed for surface expression of CD45RB and CD44 on the CD62L^high^ subset. CD45RB expression is downregulated upon antigen experience and can be used to gauge activation levels of CD4 T cells. **A.** CD45RB and CD44 on CD62L^high^ splenic CD4 T cells. **B–D.** Meth-treated animals had a higher proportion of CD45RB^high^, CD44^low^ CD4 T cells compared to controls. Meth-treated animals also exhibited lower proportions of CD45RB^low^, CD44^low^ and CD45RB^low^, CD44^high^subsets compared to controls. ***p<0.001 calculated by Mann-Whitney U Test. Bars represent mean. Data are from 2 experiments of 5 animals per treatment group per experiment.

**Figure 7:**
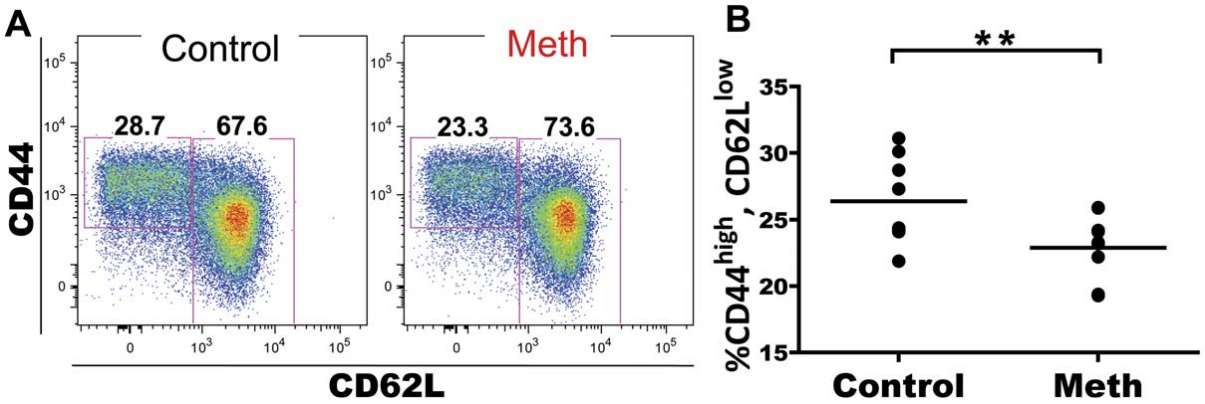
Meth treatment causes a reduction in proportion of CD62L^low^, CD44^high^ CD4 T cells in the mesenteric lymph node (MLN). MLN CD4 T cells were labeled with CD62L and CD44 to determine proportions of antigen-experienced cells. **A.** CD62L and CD44 on MLN CD4 T cells from meth and vehicle treated animals. **B.** Meth causes a reduction in proportion of CD44^high^, CD62L^low^ CD4 T cells in the MLN. **p<0.01 calculated by Mann-Whitney U Test. Bars represent mean. Data are from 2 experiments of 5 animals per treatment group per experiment.

Meth has been associated with altered T cell activation and proliferation. We investigated the role of meth on CD4 and CD8 T cells in the spleen and mesenteric lymph node (MLN) to determine if meth influences antigen-experienced and naïve subsets of T cells *in vivo*. Within the splenic CD4 T cell compartment, we observed a significant decrease in proportion of CD62L^low^, CD44^high^ antigen-experienced cells after meth treatment (p<0.0001; Fig. 5A & B). Futhermore, within this CD62L^low^, CD44^high^ subset we observed a decreased proportion of cells expressing high levels of CD27 in meth-treated mice compared to controls (p = 0.0029; Fig. 5C & D). Among CD62L^+^ CD4 T cells, we observed a significant increase in proportion of naïve CD45RB^bright^, CD44^low^ cells (p<0.0001), with significant decreases in proportion of CD45RB^low^, CD44^low^ (p = 0.0003) and CD45RB^low^, CD44^high^ cells (p = 0.0001; Fig. 6A–D). Combined, these results suggest a decrease in overall activation/antigen experience by CD4 T cells. While a similar decrease in CD62L^−^, CD44^high^ proportion was noted in the MLN (p = 0.0052; Fig. 7A & B), we did not observe differences in CD27 expression within this subset in this organ (data not shown). We also observed no differences in the CD62L^+^ population of MLN CD4 T cells after meth treatment (data not shown). In total, meth appears to inhibit CD4 T cell activation in secondary lymphoid tissue.

#### Meth causes upregulation of CD150 (SLAM) on CD226^+^ CD4 T cells

**Figure 8:**
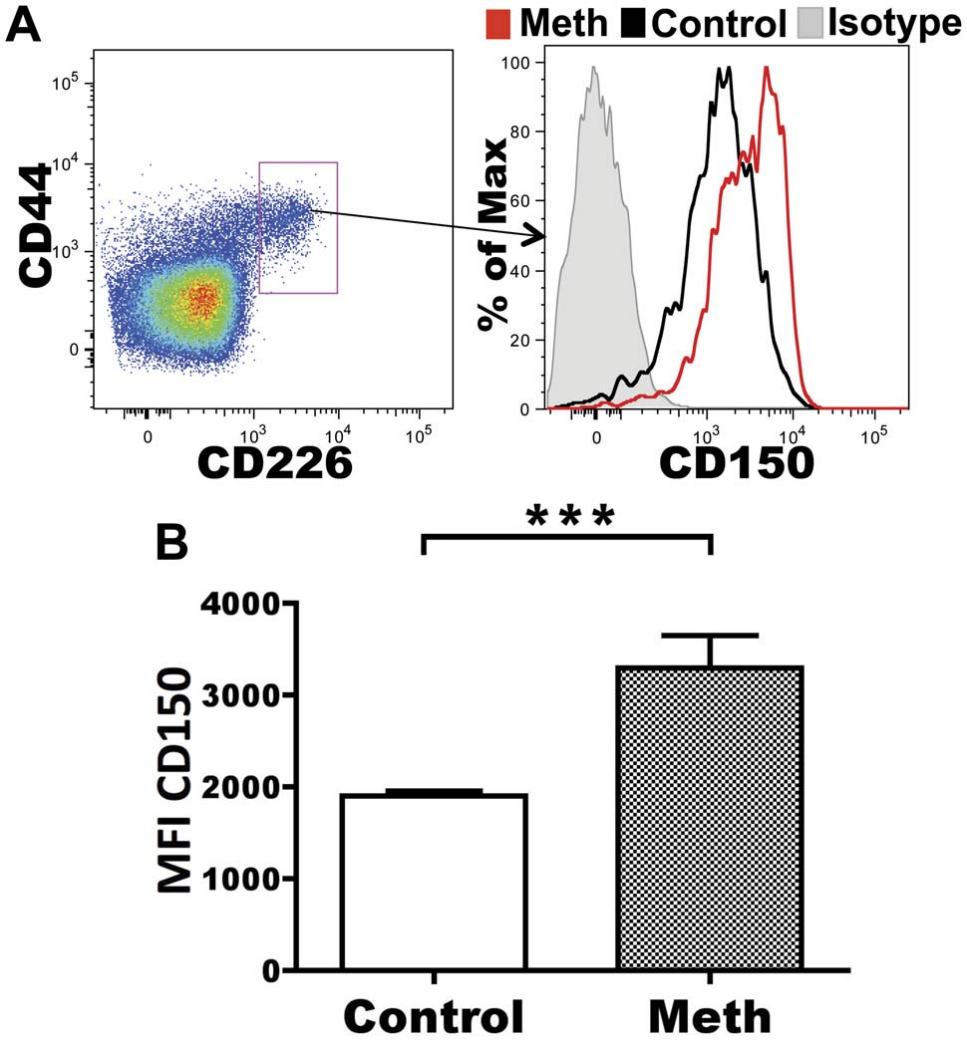
Increased expression of CD150 on CD226^+^ CD4 T cells after meth treatment. We examined the expression of the costimulatory markers CD150 and CD226 by splenic CD4 T cells after meth treatment. **A.** Representative gating and histogram showing selection of CD226^+^ CD4 T cells and increased expression of CD150 by meth treated animals compared to control. Red represents a meth-treated animal, black is vehicle-treated animal, and grey is the isotype control. **B.** CD226^+^ CD4 T cells from meth treated animals express higher levels of CD150 than those from control animals. Protein expression was calculated using mean fluorescence intensity (MFI). ***p<0.001 calculated by Mann-Whitney U Test. Error bars represent SEM. Data are from 2 experiments of 5 animals per treatment group per experiment.

Members of the signaling lymphocytic activation (SLAM) family are associated with T cell activation, and signaling through CD150 (SLAM) results in IFN-γ production, differentiation, and proliferation by T cells [47]. CD226 expression has been associated with Th1 status in murine T cells [48]. We hypothesized that meth’s negative impact on T cell activation should correspond to a decrease in CD150 expression as well as a decreased proportion of CD226-expressing CD4 T cells. To test this hypothesis, we compared CD150 MFI and proportion of CD226^+^ CD4 T cells from meth-treated and control mice. Although we observed no differences in CD226 expression after meth treatment (data not shown), we did observe significantly increased expression of CD150 on CD226^+^ CD44^+^ CD4 T cells (p<0.0001; Fig. 8A & B). The upregulation of this costimulatory molecule suggests that meth promotes the activation of specific subsets of CD4 T cells.

#### Meth causes the expansion of a KLRG1^+^ CD4 T cell subset that is not FoxP3^+^

**Figure 9:**
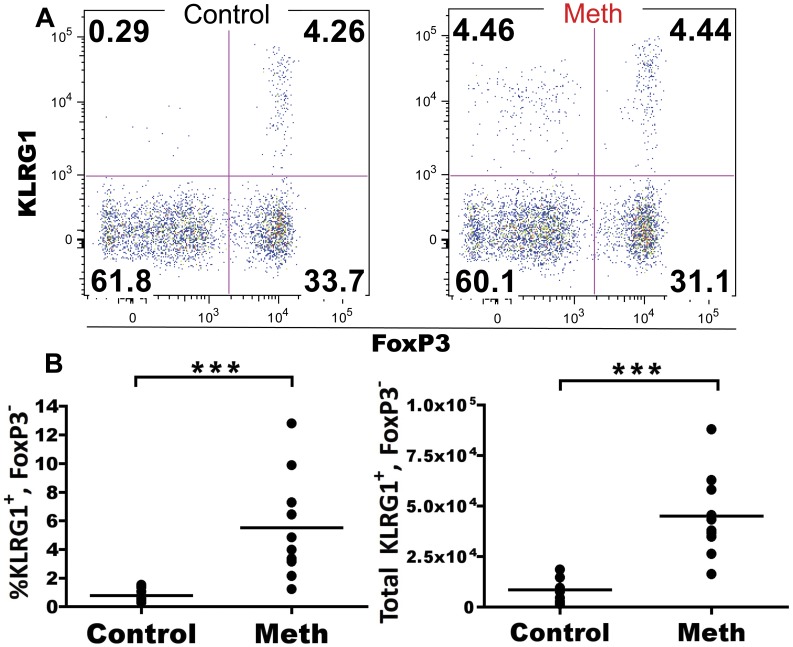
Meth promotes the expansion of splenic KLRG1^+^, FoxP3^−^ CD4 T cells. Meth-treated animals possessed an expansion of KLRG1^+^ CD4 T cells within the spleen. These cells were CD44^high^ and negative/low for FoxP3, CD25, CD62L, NKG2D, and CD45RB. **A.** Representative gating showing significant expansion of splenic KLRG1^+^, FoxP3^−^ CD4 T cells among the CD44^high^, CD45RB^low^ compartment with relatively unchanged proportions of FoxP3-expressing subsets after meth treatment. **B and C.** Proportion and absolute number of KLRG1^+^, FoxP3^−^ CD4 cells are increased with meth. ***p<0.001 calculated by Mann-Whitney U Test. Bars represent mean. Data are from 2 experiments of 5 animals per treatment group per experiment.

Regulatory T cells (T_REGS_) are fundamental in maintaining tolerance [49] and within the T_REG_ population, a potent regulatory compartment expressing KLRG1 has been reported in healthy mice [50]. To determine the effect of meth on specific subsets of T_REGs_, we labeled splenic CD4 T cells with FoxP3, CD25 (IL-2Rα), and KLRG1, with additional markers of homing and differentiation. Although we observed no significant difference in KLRG1^+/−^, FoxP3^+^, CD25^+^ CD4 T cells after meth treatment (data not shown), we did observe a significant expansion of KLRG1^+^ FoxP3^−^ CD4 T cells in proportion and absolute number after meth treatment (p<0.0001; Fig. 9A & B). These cells were CD44^high^, CD45RB^low^, CD25^low^ CD62L^low^, and NKG2D^−^ (data not shown), a profile of protein expression suggesting activation. A similar KLRG1^+^ expansion was observed in MLN, although FoxP3 staining was not performed in the MLN (data not shown). These data from spleen and MLN demonstrate that meth promotes the expansion of a CD4 T cell subset with a terminal effector phenotype, an unexpected find amidst signs of suppression.

### CD8 T Cells

#### Meth treatment results in decreased proportions of activated CD8 T cells in spleen and lymph node

**Figure 10:**
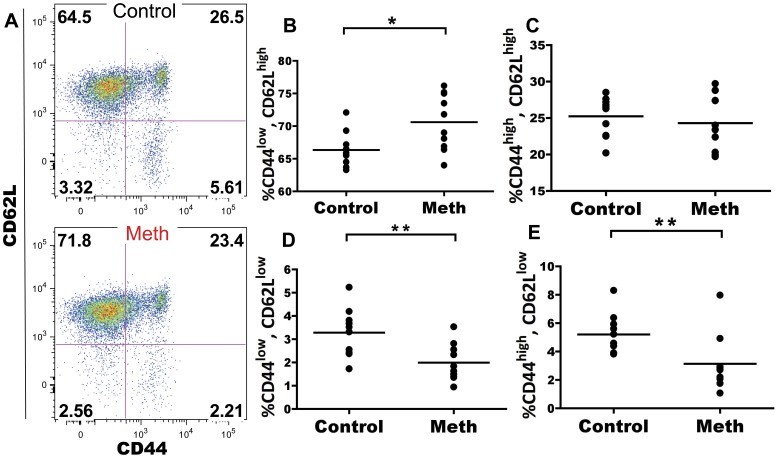
Meth-treated animals exhibit a greater proportion of naïve phenotype CD8 T cells and reduced proportions of antigen-experienced subsets within the spleen. Splenic CD8 T cells were labeled with CD62L and CD44 and classified into 4 groups according the expression of these two proteins: CD62L^high^, CD44^low^ (Naïve CD8 T cell, T_N_), CD62L^high^, CD44^high^, (central memory CD8 T cell, T_CM_), CD62L^low^, CD44^high^ (effector memory CD8 T cells, T_EM_), and CD62L^low^, CD44^low^ (acute effector CD8 T cell, T_AE_). **A.** Gating showing CD44 and CD62L expression by splenic CD8 T cells. **B–E.** After meth treatment**,** the T_N_ compartment is increased in proportion, while the T_AE_ and T_EM_ showed a decrease in proportion and T_CM_ appeared unchanged. *p<0.05, **p<0.01 calculated by Mann-Whitney U Test. Bars represent mean. Data are from 2 experiments of 5 animals per treatment group per experiment.

**Figure 11:**
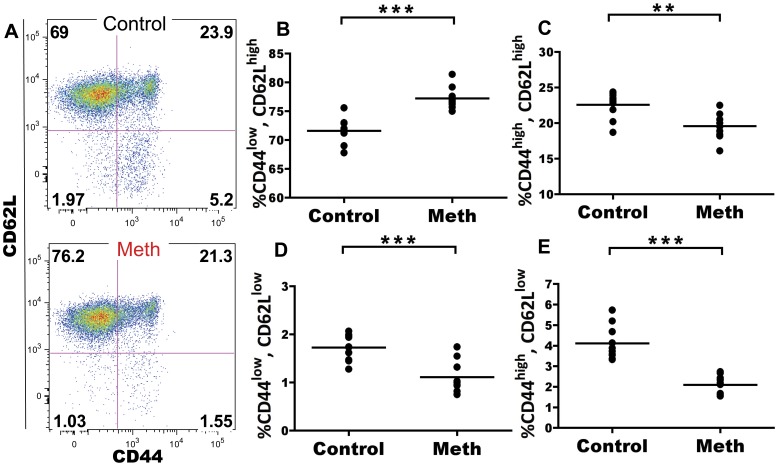
CD8 T cells from the mesenteric lymph node (MLN) also show reductions in proportion of antigen-experienced subsets with an increase in the naïve compartment. Mesenteric lymph node CD8 T cells were labeled with CD62L and CD44 and classified as in Fig. 10. **A.** Gating showing CD44 and CD62L expression by MLN CD8 T cells. **B–E.** After meth treatment**,** the T_N_ compartment is increased in proportion, while the T_AE_, T_EM_, and T_CM_ compartments all revealed a decrease in proportion. **p<0.01, ***p<0.001 calculated by Mann-Whitney U Test. Bars represent mean. Data are from 2 experiments of 5 animals per treatment group per experiment.

Naïve CD8 T cells can differentiate into cytotoxic effectors with short-lived status or long-term memory potential. Since meth-induced deficiencies in these effector subsets may leave the user more vulnerable to pathogens, we investigated meth’s ability to alter CD8 T cell subpopulations. CD8 T cells can be divided into 4 subsets depending on levels of CD62L and CD44 expression: CD62L^high^, CD44^low^ (naïve CD8 T cell, T_N_), CD62L^high^, CD44^high^, (central memory CD8 T cell, T_CM_), CD62L^low^, CD44^high^ (effector memory CD8 T cells, T_EM_), and CD62L^low^, CD44^low^ (acute/activated effector CD8 T cell, T_AE_) [51], [52]. Within the spleen, we observed a significant increase in proportion of CD8 T_N_ cells (p = 0.0185) with a decrease in proportion of T_EM_ (p = 0.0021) and T_AE_ (p = 0.0021) subsets, but no change in T_CM_ proportion (Fig. 10A–E). Similar alterations were observed in the MLN. Mesenteric lymph node CD8 T cells in meth-treated mice showed a significant increase in proportion of CD8 T_N_ cells (p<0.0001) with a decrease in proportion of T_EM_ (p<0.0001), T_AE_ (0 = 0.0011), and T_CM_ subsets (p = 0.0029; Fig. 11 A–E). From these results, it appears that meth also negatively impacts the accumulation of antigen-experienced CD8 T cells, similar to what was observed in CD4 T cells.

#### Meth treatment results in decreased numbers of KLRG1^+^ CD8 T cells in the MLN

**Figure 12:**
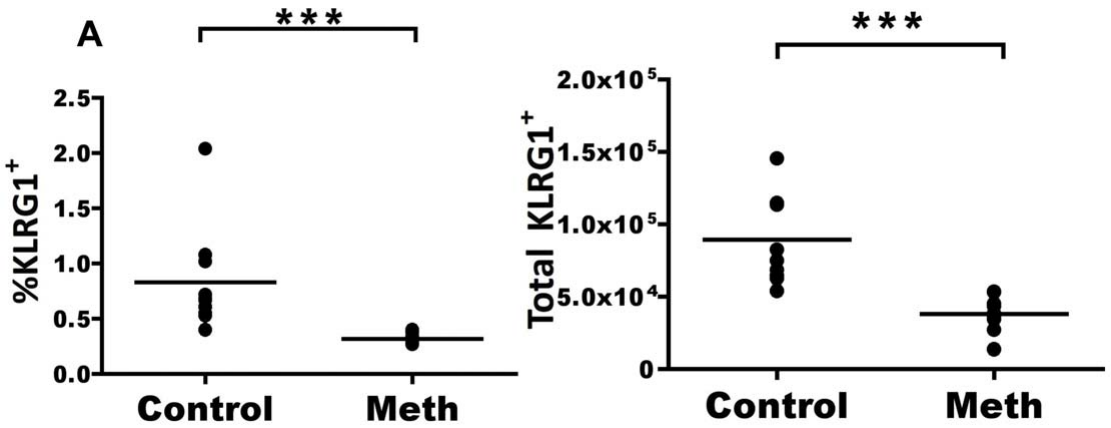
Meth causes a reduced proportion and number of KLGR1^+^ CD8 T cells in the MLN. CD8 T cells were labeled with KLRG1 to investigate whether meth impacts subsets of antigen-experienced CD8 T cells. **A.** The proportion and number of CD8 T cells expressing KLRG1 in the MLN is reduced after meth treatment. *p<0.01, ***p<0.001 calculated by Mann-Whitney U Test. Bars represent mean. Data are from 2 experiments of 5 animals per treatment group per experiment.

KLRG1 is expressed by effector CD8 T cells [53], and we postulated that meth’s disruption of antigen presenting pathways and T cell activation would also effect the profile of this marker of CD8 T cell activation. Interestingly, we observed a significant decrease in KLRG1-expressing CD8 T cells (p<0.0001) in the MLN (Figure 12 A), yet we observed no difference in KLRG1 expression by CD8 T cells in the spleen (data not shown). This reduction in the MLN adds further evidence to support the notion that meth reduces specific effector T cells and may promote weakened immunity.

**Figure 13:**
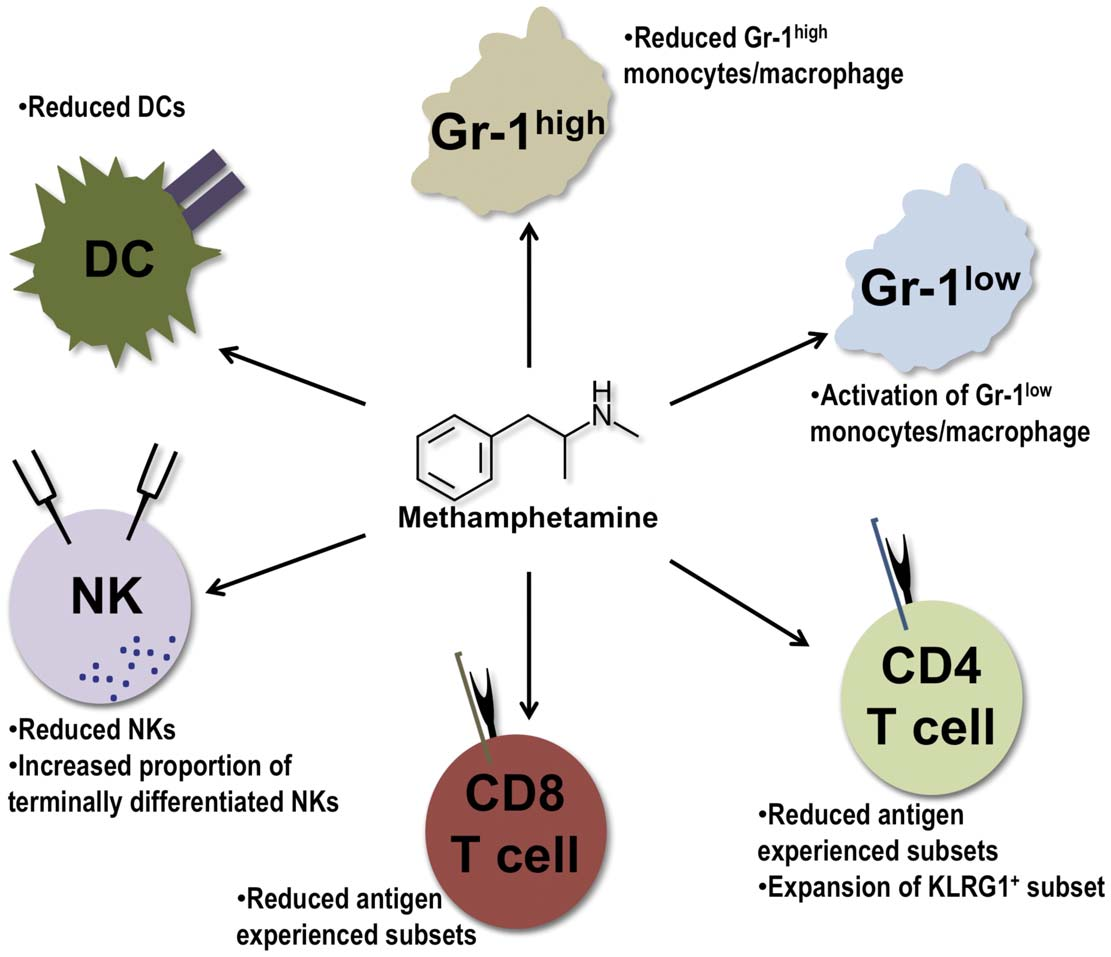
Methamphetamine impacts the innate and adaptive arms of immunity. Summary of the effects of methamphetamine on immune cell subsets based upon the observations in this report.

## Discussion

### Meth Decreases the Abundance of Splenic Innate Leukocytes

Previous studies have suggested meth has the potential to disrupt immune homeostasis and leave the user more susceptible to pathogenic challenge. With meth impacting the function of NK cells and antigen-presenting cells (APCs), a basis of innate immunity becomes weakened, and consequent and appropriate adaptive responses by T and B cells are less likely. In this study, we observed an overall decrease in both proportion and number of splenic NK cells, DCs, and Gr-1^high^ monocytes/macrophages. Meth-induced leukopenia had been previously reported by Saito et al. [37], who noted reduced numbers of leukocytes in the blood of male Slc:ddY mice at 1, 4, 24, and 48 hours after meth treatment, but did not determine which subsets were impacted by meth. Furthermore, our results showing no difference in splenic T and B cell proportion after meth treatment are in agreement with the findings of Wey et al. [31], who analyzed splenocytes from meth-treated BALB/c mice for proportion of B220, CD4 and CD8-expressing cells. While the effect of meth of promoting apoptosis [28], [39] may explain some of this reduction in cell number, it would also raise the question as to why certain subsets remain relatively unchanged after meth exposure.

The findings of Saito et al. showing leukocyte deficiencies in blood and our observed reductions of myeloid populations within the spleen may suggest a sensitivity to meth in the bone marrow, a lipid-rich environment that may store meth and have detrimental effects on bone marrow-derived leukocyte subsets. It is known that meth is lipophilic and can accumulate in solid tissue [12], [13], and it has been reported that meth can persist in bone marrow [54]–[56]. Additionally, In et al. [30] reported reduced granulocyte-macrophage colony formation in GM-CSF-treated cultures of bone marrow harvested from meth-treated CD-1 mice. For these reasons, it would be meaningful to perform additional investigations on the effects of meth on haematopoesis and the seeming imperviousness to meth by B cells, neutrophils, and Gr-1^low^ monocytes/macrophages compared to other bone marrow-derived subsets.

The immunological roles of Gr-1(Ly-6C)^low^ and Gr-1(Ly-6C)^high^ monocytes/macrophages are not completely clear, but it has been shown that Ly-6C^low^ monocytes appear to be a more stable, tissue resident and endothelium-monitoring population capable of macrophage and DC differentiation, while Ly-6C^high^ monocytes tend to home to areas of inflammation and mature to inflammatory DCs and macrophages [57]. Our observation of reduced splenic Gr-1(Ly-6C)^high^ monocytes/macrophages fits well with the reduced number of peritoneal macrophages after meth exposure reported by In et al. [32]. In their study, they utilized the intraperitoneal thioglycolate injection model of inflammation to promote macrophage accumulation in the peritoneum. This model has been shown to cause the migration of Ly-6C^high^ (but not Ly-6C^low^) monocytes to the peritoneum [57], where they ultimately mature to macrophage. The noted deficiencies in peritoneal macrophages correlates with our observation of reduced numbers of splenic precursors to this population. Indeed, the spleen has been shown to be a significant reservoir of mature monocytes and upon inflammatory signaling, to exhibit substantial decreases in monocyte numbers as cells emigrate [42].

### Perturbed Gr-1^low^ Monocytes/macrophages may be Responding to Meth-induced Cytotoxicity and Exhibiting a Tolerogenic Function

In addition to alterations in splenic abundance, our results indicate that the Gr-1(Ly-6C)^high^ and Gr-1(Ly-6C)^low^ monocyte/macrophage populations are unequally affected by meth in terms of activation. While we observed no difference in CD86 and CD80 expression by Gr-1(Ly6C)^high^ monocytes/macrophages, we did observe a significant increase in CD80 expression by Gr-1(Ly-6C)^low^ monocytes/macrophages, with no change in their CD86 expression. In addition to their role in vascular repair and monitoring [58], Ly6C^low^ monocytes/macrophages have been reported to operate as phagocytes, capable of engulfing debris from dead cells and ultimately promoting T cell proliferation [59] or self tolerance [60]. If meth is capable of inducing increased rates of apoptosis as suggested by Potula et al. [28] and Iwasa et al. [39], perhaps this population of monocytes/macrophages is responding to an accumulation of dead material and maturing to promote tolerance. Indeed, CD80 has been shown to bind PDL-1 [61], [62] and this interaction results in reduced rates of proliferation among T cells [61], [63], suggesting a tolerogenic function of CD80 in addition to it’s known role in costimulation [64]. Although CD80-CTLA-4 interactions have also been proposed to terminate effector T cell responses in the periphery [44], our data do not lend themselves to this interpretation. The Ly-6C^low^ monocytes/macrophages in our study are MHC II^−^, thus they are not presenting antigen in this manner and therefore are unlikely to be involved in specific TCR-MHC II interactions. Furthermore, the only expansion of T cells we observed was within the CD4 compartment, suggesting MHC II dependency and providing little evidence for cross presentation.

Accumulation and removal of apoptotic cells may also explain our observed decrease in CD11b expression on Ly-6C^low^ monocytes/macrophages. A recent report by Schif-Zuck et al. [65] has described a decrease in CD11b expression on “satiated” macrophages resulting from iC3b directed internalization of apoptotic cells. Although work by Talloczy et al. [33] and Martinez et al. [34] has suggested reduced phagocytic efficacy due to the basic properties of meth impacting with the acidic endosome of macrophages, findings that suggest reduced antigen-presentation effectiveness and activation, their results did not suggest *eliminated* antigen processing and internalization capabilities, rather, *attenuated*, and therefore are not in disagreement with our findings.

### Meth-induced Alterations in NK Cell Proportion and Number Suggest Functional Deficiencies

The fundamental roles performed by NK cells in viral immunity [66], tumor surveillance [67], and guiding the adaptive immune response have been revealed [68]. Consequently, a deficiency in NK cell number as well as NK cell function may lead to weakened immunity. Our observation of an increased proportion of terminally differentiated NK cells corresponds with previous reports suggesting altered activation levels of NK cells after meth exposure [30], [35]. KLRG1 expression by NK cells is almost exclusively restricted to the CD11b^high^, CD27^low^ subset [45], [46], [69], a group that exhibits lower levels of homeostatic proliferation [45], [69] and possesses lower levels of cytotoxicity than its CD27^high^ counterpart [45]. KLRG1-expressing NK cells have also been shown to produce lower levels of IFN-γ than KLRG1^−^ NK cells [70]. Our data suggest that meth causes a shift in the NK cell compartment, with a marked reduction in CD27^high^, KLRG1^−^ NK cells after meth treatment. This shift leaves the majority of NK cells in periphery with a phenotype suggesting lower responsiveness and effectiveness, a dangerous combination considering the importance of NK cells during viral infections and the increased susceptibility of meth users to several viruses.

### T Cells Exhibit Signs of Suppression as well as Activation After Meth Treatment

We have observed that meth causes a reduction of activated/antigen-experienced CD4 and CD8 T cells. We noted substantial decreases in proportion of CD44^high^, CD62L^low^ CD4 T cells after meth treatment and within this population, we observed a decrease in proportion of cells expressing CD27. Combined, these results suggest a decrease in CD4 T cell activation perhaps due to diminished antigen presentation as well as a polarization to short-lived status, as CD27 expression has been associated with longevity in T cells [71].

Within the CD62L^+^ subset of CD4 T cells, we observed a decrease in proportion CD45RB^low^/CD44^low^, and CD45RB^low^/CD44^high^ cells, with an increased proportion of CD45RB^high^/CD44^low^ cells. While the majority of naïve splenic T cells express CD45RB, after antigen experience these cells will undergo alternate exon splicing and downregulate CD45RB [72]. The observed deficiencies in CD45RB downregulation may be induced by reduced APC effectiveness as well as the reduced number of DCs observed in our experiment. Alternatively, meth may directly obstruct CD45 exon splicing in some fashion. An additional possibility is that meth promotes increased thymic output or enhanced proliferation by naïve subsets within the spleen. However, our data do not reveal an increase in splenic T cell number, nor did we find an altered CD4:CD8 ratio after meth treatment. Although we did not investigate the thymus in our study, it is probable that meth also accumulates within thymic tissue and may impact thymic selection and output. Supporting this notion, Iwasa et al. [39] observed an increased proportion of apoptotic cells in the thymus after a single injection of meth, while In et al. [30] noted an increase in proportion of CD4 T cells in the thymus with decreases in proportion CD8 and double positive (DP) T cells accompanied with reduced thymic weight after meth use, findings which justify further investigations of the effects of meth on the thymus.

CD8 T cell activation patterns were similarly affected as those of CD4 T cells. We observed reduced proportions of effector memory (T_EM_) and acute effector (T_AE_) CD8 T cells subsets in the spleen and reduced proportions of T_EM_, central memory (T_CM_), and T_AE_ subsets in the MLN, again suggesting either decreased antigen presentation and/or increased cell death among these reduced populations. These overall decreases in CD4 and CD8 T cell activation may be highly detrimental during the host’s response to pathogenic challenge. Considering that meth users are at increased risk for HIV and other viral infections, suppression of the immune adaptive response would be expected to lead to exacerbated infections. In agreement with this reasoning, meth use accompanying HIV has been associated with increased viral loads in human subjects [73].

Although our investigations revealed signs of suppression of the CD4 and CD8 T cell response, we did observe associations suggesting activation, namely, increased CD150 expression by CD226^+^ CD4 T cells and an expansion of KLRG1^+^ CD4 T cells. CD150 upregulation by T cells has been observed after concanavalin A [47] and anti-CD3 [74] stimulation, and CD150 costimulation with anti-CD3 antibodies induced increased IFN-γ prodution and proliferation compared to anti-CD3 antibody treatment alone [47]. In our analysis of the splenic CD4 T cell compartment after meth treatment, we noted an increase in CD150 expression among CD226^+^ CD4 T cells that are also CD44^high^ and CD62L^low^. This is an interesting finding considering that overall CD44^high^,CD62L^low^ numbers were decreased with meth use, thus suggesting a specific subset(s) of unknown TCR specificity is somehow activated by meth, while others are diminished.

Killer cell lectin-like receptor G1 (KLRG1) is an inhibitory C-type lectin receptor that binds to E, N, and R cadherins [75], [76] and we observed alterations in KLRG1 expression by NK cells and CD4 and CD8 T cells after meth treatment. Interestingly, we noted an expansion of KLRG1^+^ FoxP3^−^ CD4 T cells that appears surprising in the midst of signs of several signs of immune suppression. Increased KLRG1^+^ CD4 T cells have been reported after *Toxoplasma gondii* infection [70] and *Mycobacterium tuberculosis* infection [77], while KLRG1^+^ CD4 T cells were found to be unresponsive to TCR-induced proliferation [50] and produced high levels of IFN-γ and TNF-α after peptide stimulation [77]. Beyersdorf et al. [50] also observed that a portion of FoxP3^+^ CD4 T cells coexpressed KLRG1 and CD152, were CD25^+/−^, and that KLRG1^+^, CD25^+/−^ CD4 T cells were capable of reducing cellular proliferation of anti-CD3 stimulated naïve CD4 T cells in the presence of APC, as well as limiting H^3^ thymidine uptake by TCR-stimulated naïve CD4 T cells. From these studies, we can conclude that there are at least two main populations of KLRG1^+^ CD4 T cells in mice: one which represents a terminally differentiated effector, and another which is a potent T_REG_. While we did observe KLRG1^+^ expression amongst FoxP3^+^ CD4 T cells and these cells were CD25^+/−^, we did not find a difference in proportion or number of any FoxP3-expressing CD4 T cell subset after meth treatment. Phenotypically, our KLRG1^+^ CD4 T cell subset fits into an effector/memory phenotype, being CD62L^−^, CD45RB^low^, CD25^−^, FoxP3^−^ and CD44^high^. However, future studies must be conducted to determine the functional attributes of this population and to determine its Th status. 1558–1565.

### Conclusions

Our results demonstrate that meth impacts several leukocyte populations (Fig. 13). Indeed, meth’s role in inducing apoptosis and inhibiting APC function may have tremendous effects on T cell memory populations as well as on T cell activation, as our results and those of others have suggested. Furthermore, the combined effects of a decrease of Gr-1^high^ monocytes/macrophages, DCs, and NK cell numbers, with a proportional increase of a less-responsive NK cell subset may leave the user less able to initiate a sufficient immune response and ultimately reduce the effectiveness of the adaptive response. Although our data reveal specific descriptive alterations induced by meth and suggest a reduced ability to respond efficiently to pathogens, additional investigations must focus on how the innate and adaptive arms are impacted in the face of viral and bacterial challenge concurrent with meth exposure. As meth addicts are at risk for increased rates of sexually transmitted diseases (STDs) and blood borne pathogens due to behavioral responses and drug administration routes, these investigations would be meaningful in revealing which mechanisms promote increased susceptibility to infections as suggested by this work and previous studies.
